# Genomic characterization of SNW-1, a novel prophage of the deep-sea vent chemolithoautotroph *Sulfurimonas indica* NW79

**DOI:** 10.1590/1678-4685-GMB-2023-0355

**Published:** 2024-07-29

**Authors:** Xiaofeng Li, Ruolin Cheng, Chuanxi Zhang, Zongze Shao

**Affiliations:** 1 Ningbo University, Institute of Plant Virology, State Key Laboratory for Managing Biotic and Chemical Threats to the Quality and Safety of Agro-Products, Ningbo, China.; 2 Key Laboratory of Biotechnology in Plant Protection of Ministry of Agriculture and Zhejiang Province, Ningbo, China.; 3 Third Institute of Oceanography, Ministry of Natural Resources, Key Laboratory of Marine Genetic Resources, Xiamen, China.; 4 State Key Laboratory Breeding Base of Marine Genetic Resource, Xiamen, China.

**Keywords:** Sulfurimonas indica, Campylobacterota, prophage, phylogenetic analysis.

## Abstract

The globally widespread genus *Sulfurimonas* are playing important roles in different habitats, including the deep-sea hydrothermal vents. However, phages infecting *Sulfurimonas* have never been isolated and characterized to date. In the present study, a novel prophage SNW-1 was identified from *Sulfurimonas indica* NW79. Whole genome sequencing resulted in a circular, double-stranded DNA molecule of 37,096 bp with a mol% G+C content of 37. The genome includes 64 putative open reading frames, 33 of which code for proteins with predicted functions. Presence of hallmark genes associated with *Caudoviricetes* and genes involved in lysis and lysogeny indicated that SNW-1 should be a temperate, tailed phage. Phylogenetic and comparative proteomic analyses suggested that *Sulfurimonas* phage SNW-1 was distinct from other double stranded DNA phages and might represent a new viral genus.

The genus *Sulfurimonas* within *Campylobacterota* (formerly *Epsilonproteobacteria*) (Waite et al., 2017, 2018) are widespread in a variety of marine and terrestrial habitats, such as hydrothermal vent fields, pelagic redoxclines, coastal sediments, oil reservoirs, groundwater systems and sulfidic springs (Han and Perner, 2015). They are able to grow chemolithoautotrophically with different electron donors including sulfide, elemental sulfur, thiosulfate and hydrogen (Wang et al., 2020), playing important roles in the oxidative part of the sulfur cycle. To date, the genus contains 13 species with validly published names, and 5 of them were isolated from deep-sea hydrothermal vent environments (Inagaki et al., 2003; Takai et al., 2006; Hu et al., 2021; Wang *et al*., 2021a, b). Recently, we obtained a novel strain, *Sulfurimonas* sp. NW79, from a deep-sea hydrothermal vent in the Carlsberg Ridge of Northwest Indian Ocean. It shared the highest 16S rRNA gene sequence similarity (99.09%) with *S. indica* NW8N (Hu *et al*., 2021). Whole-genome sequencing of the strain NW79 revealed the presence of a putative prophage region. Here, we focus on presenting the genomic characterization of the novel prophage SNW-1. To our knowledge, this is the first report of a phage infecting a bacterium of the genus *Sulfurimonas*. 

The host bacterial strain NW79 was grown in MMJS liquid medium (Hu et al., 2021) at 28 °C and then logarithmic-phase bacterial cultures were treated with 1 μg/mL mitomycin C for 18 hours. Following incubation, the phage lysate was collected, filtered, and concentrated by polyethylene glycol (PEG) precipitation. Genomic DNA of the phage and the host bacteria was extracted using a phage DNA isolation kit (Yuanye Bio-Technology Co. Ltd., Shanghai, China) and a SBS extraction kit (SBS Genetech Co. Ltd., Shanghai, China), respectively, following the manufacturer’s instructions. Whole genome sequencing of the host bacteria was performed on Illumina Hiseq PE150 platform (Illumina Inc., San Diego, CA, USA), and the raw reads were trimmed and quality filtered using the fastp software (Chen et al., 2018). In addition, DNA samples were prepared for long-read sequencing with the Oxford Nanopore Technologies (ONT) ligation library preparation kit according to the manufacturer’s standard protocol, and the libraries were sequenced by the ONT MinION sequencer. Hybrid *de novo* assembly of Illumina and Nanopore reads was then performed using SPAdes v3.14.0 (Bankevich et al., 2012). Putative open reading frames (ORFs) were predicted using the Prokka pipeline (Seemann, 2014) and verified by the RAST annotation server (http://rast.nmpdr.org/). Putative proteins were annotated by homology searching against the NCBI’s non-redundant protein database (December, 2022) using BLASTp (E-values < 10^−5^) (Camacho et al., 2009). HMM search against the Pfam (release 31.0) (Finn et al., 2013) and Virus Orthologous Groups (VOG, https://vogdb.org/) databases was also performed to identify the protein functional domains. The annotated sequence was visualized using DNAPlotter (Carver et al., 2008).

As a result, the SNW-1 prophage genome was assembled into a single contiguous sequence (contig) of 37,217 bp with direct terminal repeats. The contig ends were then joined at the overlapping region, producing a circular genome sequence with a length of 37,096 bp. It has a G+C content of 37%, which is similar to that of the host bacterial genome. A total of 64 ORFs were predicted, 40 (62.5%) of which were located on the negative strand, while 20 were located on the positive strand. ATG was the predominant start codon (59 ORFs), but there were also a few ORFs with GTG or TTG as alternative start codons. Fifty-five putative ORFs showed similarities to sequences in the public database, and 33 of them were assigned a predicted function (Table S1). No tRNA or rRNA genes were identified in the genome. Based on these annotations, the phage genes were classified into five main functional groups: structural component/assembly, replication/transcriptional regulators, DNA packaging, lysogeny and lysis (Figure 1). 

**Figure 1-:**
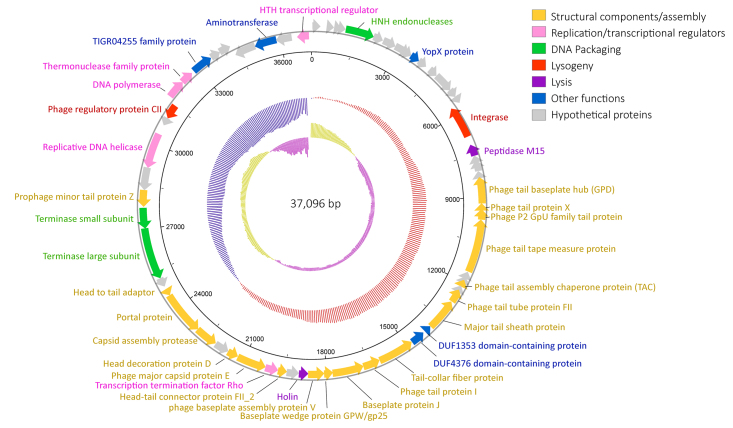
Annotated genome map of Sulfurimonas phage SNW-1. The predicted ORFs are represented by colored blocks with arrows. The GC skew is indicated in the inner circle in yellow and purple. The GC content is shown in red and blue.

Most of the predicted proteins showed highest amino acid identity to proteins from *Campylobacterota* rather than phage, suggesting the presence of prophages in these genomes. Nineteen proteins were predicted to be structural components or involved in phage assembly (Figure 1, Table S1), including the major capsid protein (ORF42), head decoration protein (ORF43), capsid assembly protease (ORF45), portal protein (ORF46), tape measure protein (ORF24), head-tail joining proteins (ORF40, ORF47), tail proteins (ORF21, ORF22, ORF23, ORF27, ORF29, ORF30, ORF33, ORF34, ORF51) and baseplate proteins (ORF35, ORF36, ORF37). Like many other temperate phages, the longest ORF in SNW-1 genome encodes the tape measure protein, which determines the phage tail length (Katsura, 1987). Interestingly, the head-related proteins were more similar to putative proteins from *Sulfuricurvum* sp. IAE1, while tail-related proteins resembled those from *Sulfurimonas* sp. UBA12504, implying exchange of blocks of genes during evolution. 

Putative proteins that reflect the temperate nature of SNW-1 were detected, including integrase (ORF16) and the phage regulatory protein CII (ORF55). The phage integrase promotes site-specific recombination of phage and host genomes and the regulatory protein CII is involved in the establishment of lysogeny (Rajamanickam and Hayes, 2018). For most double-stranded DNA phages, two proteins are required for efficient host lysis: the endolysin and the holin (Young, 1992). The ORF38 was predicted to encode a phage holin family protein, but no endolysin homolog was identified in SNW-1 genome. To determine the lysogenic status of SNW-1, we used the Prophage Tracer (Tang et al., 2021) to detect the bacterial (attB) and phage (attP) att sites. Sequencing reads were mapped to the assembled genome of *S. indica* NW79. Surprisingly, no overlapping split-read alignments were identified, suggesting that the phage was nonintegrated. We cannot exclude the possibility that the phages are in their lytic cycle, but as the genome coverage of phage SNW-1 is just slightly higher than that of its host, it is more likely that SNW-1 exists as an extrachromosomal prophage.

Several proteins related to phage DNA packaging were predicted, including a HNH endonuclease family protein (ORF5) and two terminase subunits (ORF49, ORF50). Phage terminase is responsible for cleaving the replicated genome concatemer into single copies, and the HNH protein cofactor is required for a large number of terminases (Kala et al., 2014). In addition, the phage portal protein was also involved in packaging DNA into proheads (Rao and Feiss, 2008). 

The presence of head and tail structural genes indicates that SNW-1 belongs to the tailed phage class *Caudoviricetes*. To investigate the relationships between phage SNW-1 and other tailed phages, phylogenetic analysis of the terminase large subunit (*TerL,* ORF49) gene was performed. Alignments of related protein sequences were generated using MUSCLE (Robert and Edgar, 2004) and were trimmed by TrimAl v1.2 (Capella-Gutiérrez et al., 2009). A Maximum likelihood (ML) tree was inferred using IQ-TREE2 (Minh et al., 2020), and robustness of the tree was evaluated by analyzing 1000 ultrafast bootstrap replicates. TerL sequences from phages with experimentally determined packaging mechanisms were selected as references. The final tree was visualized with FigTree v1.4.4 (http://tree.bio.ed.ac.uk/software/figtree/). 

The phylogenetic tree of *TerL* showed that the terminase of SNW-1 was clustered with proteins from other *Campylobacterota* but was distantly related with known phages isolated from *Campylobacterota* (Figure 2). It belonged to the 5′ cos phage group represented by *Escherichia* virus Lambda. During packaging, the phage terminase recognize and cut a specific site (cos site), generating fixed DNA termini with 5′ cohesive ends (Roos et al., 2007). To confirm this, we used the PhageTerm (Garneau et al., 2017) to determine the physical termini of SNW-1 genome. Clean reads were mapped onto the SNW-1 sequence, producing a coverage plot resemble those of 5′cos phage (Figure S1). This is consistent with the packaging strategy deduced from phylogeny of the *TerL* gene. The predicted termini consist of 5′ single-stranded cohesive overhangs of 12 bases (27,921-27,932 nt, AGTGCATAGCCC), which overlap the start codon of the terminase small subunit gene. The putative cos site has a higher read coverage, but reads that cross the cos site were also detected, indicating the presence of both linear and circular phage genomes. 

**Figure 2 -:**
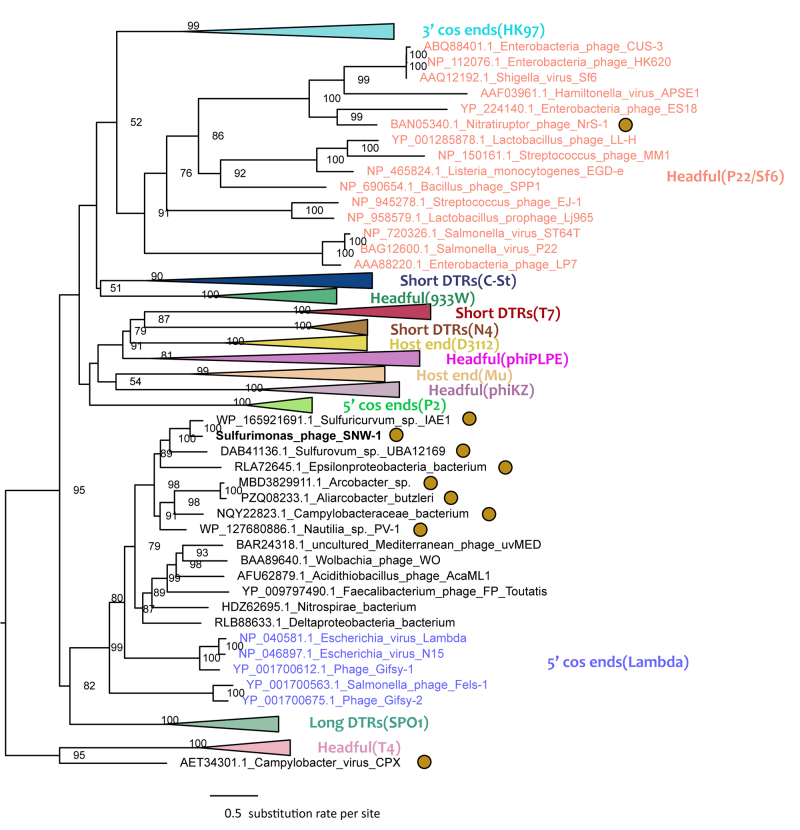
A maximum likelihood tree of the *TerL* gene based on amino acid sequences. Reference sequences from phages with experimentally determined packaging strategy were selected based on previously published studies (Bai et al., 2019), and were colored according to their packaging strategies. Abbreviations: COS, cohesive ends; DTR, direct terminal repeats. Bootstrap support values calculated from 1000 replicates are shown at the nodes. Sequences from *Campylobacterota* are indicated by brown circles.

We also generated a viral proteomic tree (Figure 3) based on genome-wide similarities using the ViPTree web server (Nishimura et al., 2017). Genomic similarity scores (S_G_) between SNW-1 and other reported prokaryotic double-stranded DNA viruses were calculated and the genomic distance matrix was used to produce the proteomic tree with BIONJ. The results showed that SNW-1 is clustered with several myoviruses (Faecalibacterium phage FP_Toutatis, Fusobacterium phage Funu1 and Vibrio phage X29) and a siphovirus (Bacteriophage Lily). However, the S_G_s of SNW-1 to these phages are quite low (0.028-0.051), indicating that it may represent a new taxon. To further determine the taxonomic position of SNW-1, a whole-genome phylogenetic tree at the nucleic acid level was inferred using the Genome-BLAST Distance Phylogeny method through VICTOR (Meier-Kolthoff and Göker, 2017), and the taxonomic classification of phages at both genus and family level was evaluated by OPTSIL (Göker *et al*., 2009). The VICTOR tree and OPTSIL taxon prediction indicated that SNW-1 belonged to the same family with Fusobacterium phage Funu1 but was a member of a new genus (Figure S2). It is well recognized now that the families *Myoviridae*, *Siphoviridae* and *Podoviridae* are not monophylic, and in the latest ICTV taxonomy these families have been abolished as well as the order *Caudovirales* (Turner et al., 2023). Fusobacterium phage Funu1 was classified as a myovirus, but now it represents an unassigned species of *Caudoviricetes*. Phage taxonomy is undergoing a profound change, as plenty of new, genome-based families will been defined. Thus, it is difficult to clarify the taxonomic status of SNW-1 at this moment. 

**Figure 3 -:**
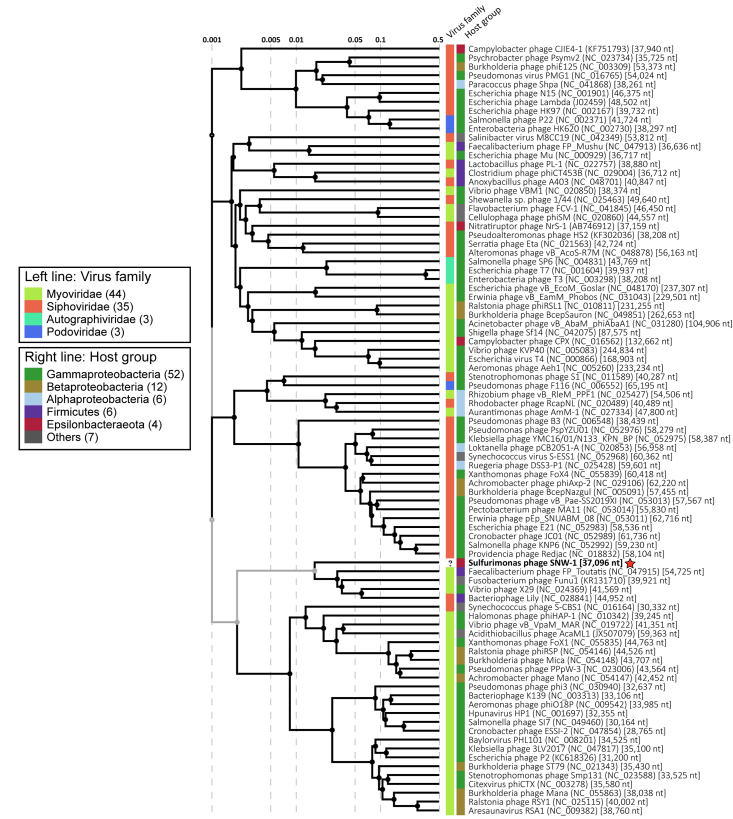
A proteomic tree of SNW-1 and related phages generated by ViPTree. The right and left lines represent the classification of the phages at the host group and family level, respectively. The phage SNW-1 is indicated by a red star.

In conclusion, analysis of genomic sequence suggested that the Sulfurimonas phage SNW-1 did not show significant similarity to any previously known tailed viruses and was distinct from reported phages of *Campylobacterota*. Further studies on the biological characteristics of the phage will provide new insight into the host-phage interactions in this widespread, ecologically important genus. 

## Supplementary material

The following online material is available for this article:

Table S1 - ORF annotations of the Sulfurimonas phage SNW-1 genome

Figure S1 - Predicted termini position of SNW-1 genome

Figure S2 - Whole-genome phylogenetic tree of SNW-1 and related phages
