# Supplementary material for: Genomic characterization of SNW-1, a novel prophage of the deep-sea vent chemolithoautotroph Sulfurimonas indica NW79
Source: Genet Mol Biol. 2024 Jul 29;47(2):e20230355. doi: 10.1590/1678-4685-GMB-2023-0355 (PMC11290706; doi:10.1590/1678-4685-GMB-2023-0355)
Supplement: Table S1 - [file 1415-4757-GMB-47-2-e20230355-s1.pdf]

Genetics and Molecular Biology

https://doi.org/10.1590/1678-4685-GMB-2023-0355

Supplementary Material to "Genomic characterization of SNW-1, a novel prophage of the deep-sea vent chemolithoautotroph Sulfurimonas indica NW79"

Table S1 - ORF annotations of the Sulfurimonas phage SNW-1 genome.

ORF	Length(aa)	Top BLAST hit	Identity%	BLAST e-value	Pfam annotation	Pfam e-value	VOG annotation	VOG e-value
ORF1	88	WP_132812355.1 hypothetical protein [Sulfuricurvum sp. IAE1]	50	3.55E-22	-	-	-	-
ORF2	72	-	-	-	-	-	-	-
ORF3	50	MBL4730763.1 hypothetical protein [Sulfurimonas sp.]	47.059	1.45E-08	-	-	-	-
ORF4	48	-	-	-	-	-	-	-
ORF5	317	HAC70149.1 hypothetical protein [Aliarcobacter skirrowii]	40.741	4.78E-94	-	-	REFSEQ HNH protein	2.70E-14
ORF6	109	-	-	-	-	-	-	-
ORF7	145	-	-	-	-	-	-	-
ORF8	164	OGS70796.1 hypothetical protein A3F91_09795 [Flavobacteria bacterium RIFCSPLOWO2_12_FULL_35_11]	50.323	4.82E-38	-	-	-	-
ORF9	46	-	-	-	-	-	-	-
ORF10	104	WP_043063261.1 YopX family protein [Aneurinibacillus migulanus]	36.8	8.33E-18	YopX protein	1.10E-20	sp|O34401|YOPX_BACSU SPBc2 prophage-derived uncharacterized protein YopX	3.70E-27
ORF11	101	-	-	-	-	-	-	-
ORF12	101	MBL1294488.1 hypothetical protein [Thiotrichales bacterium]	50	2.44E-15	-	-	-	-

ORF13	217	RLA80366.1 hypothetical protein DRG78_11110 [Epsilonproteobacteria bacterium]	47.964	3.85E-47	-	-	REFSEQ hypothetical protein	1.10E-31
ORF14	94	MCE5226491.1 hypothetical protein [Porphyromonadaceae bacterium]	35.484	3.78E-09	-	-	-	-
ORF15	81	MBD3843380.1 hypothetical protein [Campylobacterales bacterium]	67.568	8.05E-31	-	-	-	-
ORF16	378	WP_129106936.1 site-specific integrase [Halarcobacter bivalviorum]	72.751	0	Phage integrase family	7.10E-22	sp|O10330|VLF1_NPVOP Very late expression factor 1	1.60E-46
ORF17	135	AUR86027.1 peptidase M15 [Vibrio phage 1.081.O._10N.286.52.C2]	42.446	2.40E-28	-	-	REFSEQ hypothetical protein	1.40E-20
ORF18	75	WP_046996071.1 hypothetical protein [Aliarcobacter butzleri]	49.333	3.78E-18	-	-	-	-
ORF19	127	WP_066165798.1 hypothetical protein [Aliarcobacter cryaerophilus]	31.579	2.23E-13	-	-	-	-
ORF20	66	MBT8349090.1 hypothetical protein [Sulfurovum sp.]	53.333	9.20E-15	-	-	-	-
ORF21	309	DAB30689.1 TPA: phage tail protein [Sulfurimonas sp. UBA12504]	71.273	6.22E-149	Phage tail baseplate hub (GPD)	4.90E-16	sp|P10312|BPD_BPP2 Probable baseplate hub protein	6.10E-49
ORF22	69	DAB30688.1 TPA: phage tail protein [Sulfurimonas sp. UBA12504]	69.118	6.07E-25	Phage Tail Protein X	4.90E-16	-	-
ORF23	121	MCF6202028.1 phage tail protein [Hydrogenimonas sp.]	50.413	2.08E-35	Phage P2 GpU	2.30E-25	-	-
ORF24	627	DAB30686.1 TPA: phage tail tape measure protein [Sulfurimonas sp. UBA12504]	54.014	0	Phage-related minor tail protein	1.30E-28	sp|B0ZSH1|TMP_BPHA1 Probable tape measure protein	2.30E-18
ORF25	59	-	-	-	-	-	REFSEQ hypothetical protein	3.80E-17
ORF26	38	-	-	-	-	-	-	-
ORF27	75	NPA64850.1 phage tail assembly protein [Epsilonproteobacteria bacterium]	48.529	4.69E-10	Phage tail assembly chaperone proteins, E, or 41 or 14	9.10E-15	-	-
ORF28	52	-	-	-	-	-	-	-
ORF29	160	DAB30683.1 TPA: hypothetical protein CFH84_02720 [Sulfurimonas sp. UBA12504]	52.83	2.00E-51	Phage tail tube protein FII	9.60E-27	sp|P22502|TUBE_BPP2 Tail tube protein	2.50E-23

ORF30	377	DAB30682.1 TPA: phage tail protein [Sulfurimonas sp. UBA12504]	84.881	0	-	-	-	-
ORF31	84	QKF62935.1 DUF1353 domain-containing protein [Campylobacter mucosalis]			Protein of unknown function (DUF1353)	3.00E-19	REFSEQ hypothetical protein	4.10E-22
ORF32	170	WP_127680841.1 DUF4376 domain-containing protein [Nautilia sp. PV-1]	42.86	2.00E-16			REFSEQ hypothetical protein	7.60E-23
ORF33	430	WP_236891183.1 phage tail protein [Desulfoluna sp. ASN36]	35.208	1.12E-61	Phage tail-collar fibre protein	3.80E-57	REFSEQ putative tail fibers protein	2.70E-60
ORF34	201	DAB30676.1 TPA: phage tail protein I [Sulfurimonas sp. UBA12504]	44.872	3.81E-39	Phage tail protein (Tail_P2_I)	5.40E-23	sp|P26701|BPI_BPP2 Baseplate protein I	2.10E-26
ORF35	367	WP_092913056.1 baseplate J/gp47 family protein [Hydrogenimonas thermophila]	48.189	3.27E-102	Baseplate J-like protein	3.50E-30	sp|P51767|BPJ_BPP2 Baseplate protein J	3.40E-31
ORF36	103	DAB30674.1 TPA: baseplate assembly protein [Sulfurimonas sp. UBA12504]	56.311	1.04E-30	-	-	sp|P09425|BP25_BPT4 Baseplate wedge protein gp25	7.80E-13
ORF37	187	MBD3842684.1 phage baseplate assembly protein V [Campylobacterales bacterium]	49.223	2.19E-51	Type VI secretion system, phage-baseplate injector	1.80E-24	sp|P31340|SPIKE_BPP2 Spike protein	3.80E-18
ORF38	108	WP_201335168.1 MULTISPECIES: phage holin family protein [Nitratiruptor phage NrS-2]	57.282	1.10E-42	LydA holin phage, holin superfamily III	3.00E-24	sp|Q3T4L9|HOLIN_BPPRD Holin	1.70E-26
ORF39	142	WP_108062703.1 hypothetical protein [Poseidonibacter lekithochrous]	38.129	7.91E-23	-	-	-	-
ORF40	104	MWL49931.1 phage tail protein [Escherichia coli] MWU51436.1 phage tail protein [Escherichia coli] MWU56304.1 phage tail protein [Escherichia coli]	28.571	4.00E-14	Phage Head-Tail Attachment	8.20E-05	-	-
ORF41	150	WP_132812336.1 Rho termination factor N-terminal domain-containing protein [Sulfuricurvum sp. IAE1]	49.032	6.47E-37	-	-	-	-
ORF42	343	TDA64282.1 major capsid protein [Sulfuricurvum sp. IAE1]	70.554	0	Phage major capsid protein E	8.30E-85	sp|Q6KGI8|CAPSD_BPFO1 Major capsid protein	3.70E-60
ORF43	126	WP_132812338.1 head decoration protein [Sulfuricurvum sp. IAE1]	64.516	9.82E-44	Bacteriophage lambda head decoration protein D	8.50E-21	REFSEQ hypothetical protein	2.10E-25
ORF44	169	WP_132812339.1 hypothetical protein [Sulfuricurvum sp. IAE1]	50.877	1.64E-45	-	-	-	-

ORF45	264	WP_132812340.1 S49 family peptidase [Sulfuricurvum sp. IAE1]	74.621	1.69E-146	Peptidase family S49	3.80E-25	sp|P03711|SCAF_LAMBD Capsid assembly protease C	2.00E-56
ORF46	519	TDA64286.1 phage portal protein [Sulfuricurvum sp. IAE1]	70.898	0	Phage portal protein, lambda family	5.60E-110	sp|P36272|PORTL_BPP21 Portal protein B	3.00E-121
ORF47	77	DAV65442.1 TPA: MAG TPA: head to tail adaptor [Myoviridae sp.]	56	6.00E-15			-	-
ORF48	108	MCD6432953.1 hypothetical protein [Sulfurimonas sp.]	50.495	6.95E-24	-	-	-	-
ORF49	611	WP_165921691.1 phage terminase large subunit family protein [Sulfuricurvum sp. IAE1]	78.253	0	Phage terminase large subunit (GpA)	3.10E-166	REFSEQ hypothetical protein	3.60E-16
ORF50	243	WP_090453434.1 terminase small subunit [Pseudomonas jessenii] SAMN04490187_2151 [Pseudomonas jessenii]	27.014	5.45E-08	-	-	-	-
ORF51	195	WP_108062684.1 phage tail protein [Poseidonibacter lekithochrous]	30.366	6.18E-17	Prophage minor tail protein Z (GPZ)	5.50E-05	REFSEQ hypothetical protein	1.50E-13
ORF52	266	MBL4730771.1 hypothetical protein [Sulfurimonas sp.] PHQ90122.1 hypothetical protein COB42_05770 [Sulfurimonas sp.]	55.426	1.58E-94	-	-	-	-
ORF53	414	RUM61962.1 replicative DNA helicase [Sulfurimonas sp.]	32.971	3.64E-31	DnaB-like helicase N terminal domain	6.70E-17	sp|P04530|VG41_BPT4 ATP-dependent helicase 41	1.10E-34
ORF54	92	PHR57269.1 hypothetical protein COA44_06105 [Arcobacter sp.]	53.409	7.72E-24	-	-	-	-
ORF55	169	WP_165773049.1 phage regulatory CII family protein [Malaciobacter canalis]	56.303	1.30E-29	Phage regulatory protein CII (CP76)	4.50E-13		
ORF56	207	QDT80173.1 DNA polymerase III subunit epsilon [Gimesia maris]	44.608	2.93E-59	-	-	REFSEQ hypothetical protein	8.20E-25
ORF57	143	RLA80130.1 thermonuclease family protein [Epsilonproteobacteria bacterium]	60.417	1.09E-55	Staphylococcal nuclease homologue	6.40E-09	REFSEQ hypothetical protein	9.00E-25
ORF58	237	WP_079580153.1 TIGR04255 family protein [Malaciobacter marinus]	42.478	2.22E-48	-	-	-	-
ORF59	111	WP_079580152.1 hypothetical protein [Malaciobacter marinus]	56.164	1.19E-15	-	-	-	-
ORF60	129	WP_079580151.1 hypothetical protein [Malaciobacter marinus]	60.15	3.59E-44	-	-	-	-

ORF61	248	DAX63747.1 TPA: MAG TPA: Protein of unknown function (DUF3037) [Myoviridae sp.]	27.935	9.00E-19	-	-	-	-
ORF62	260	DAY43561.1 TPA: MAG TPA: aminotransferase [Siphoviridae sp.]	35.769	2.74E-40	HipA-like kinase	9.70E-13	-	-
ORF63	205	PHR57262.1 hypothetical protein COA44_06070 [Arcobacter sp.]	30.69	3.00E-23	-	-	-	-
ORF64	138	WP_132812354.1 LexA family transcriptional regulator [Sulfuricurvum sp. IAE1]	43.662	1.44E-11	Helix-turn-helix	2.80E-08	-	-
