# Supplementary material for: Genomic characterization of SNW-1, a novel prophage of the deep-sea vent chemolithoautotroph Sulfurimonas indica NW79
Source: Genet Mol Biol. 2024 Jul 29;47(2):e20230355. doi: 10.1590/1678-4685-GMB-2023-0355 (PMC11290706; doi:10.1590/1678-4685-GMB-2023-0355)
Supplement: Figure S2 - [file 1415-4757-GMB-47-2-e20230355-s3.pdf]

Genetics and Molecular Biology

https://doi.org/10.1590/1678-4685-GMB-2023-0355

Supplementary Material to "Genomic characterization of SNW-1, a novel prophage of the deep-sea vent chemolithoautotroph Sulfurimonas indica NW79"

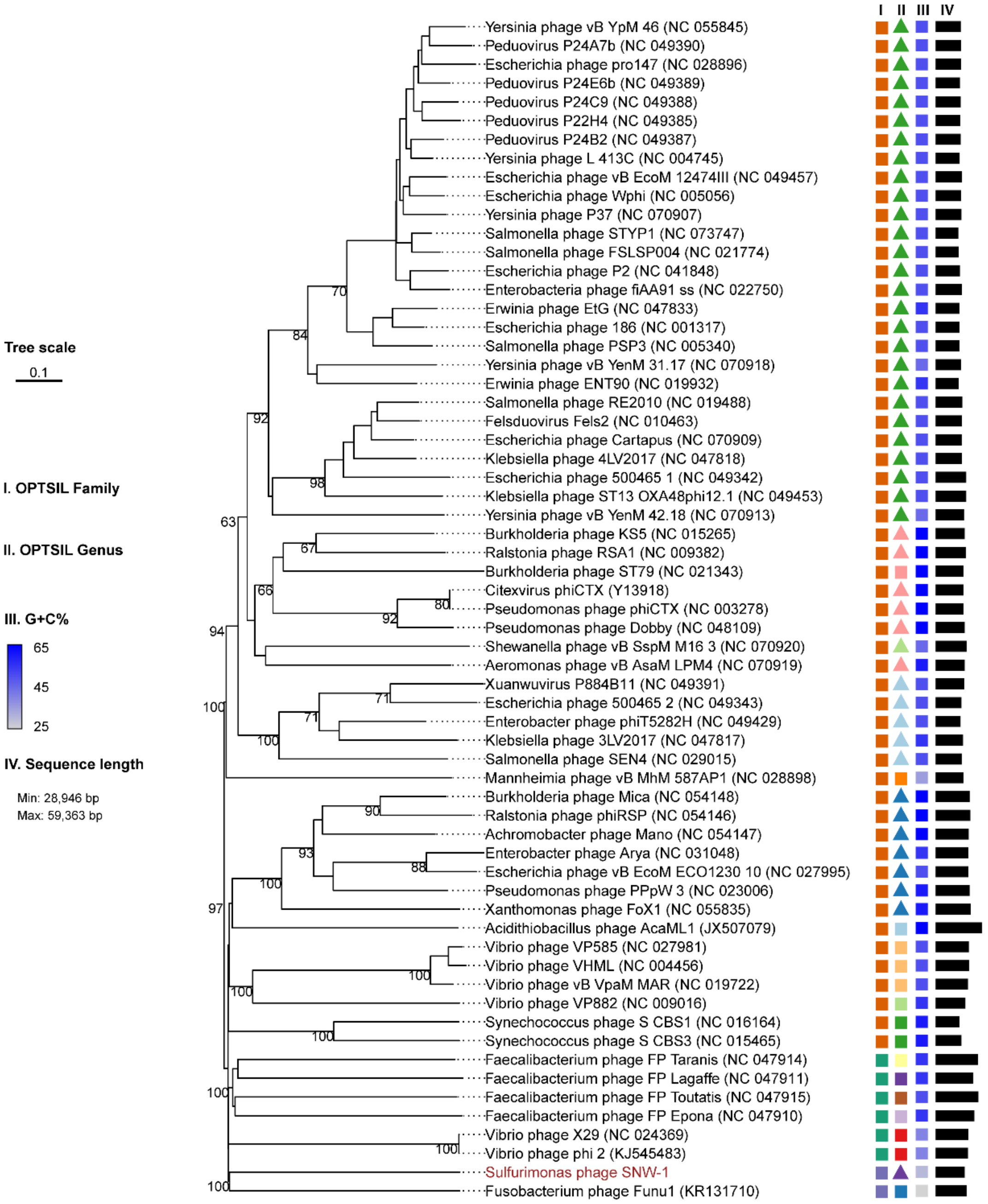

Figure S2 - Whole-genome phylogenetic tree of SNW-1 and related phages. The tree is constructed by VICTOR with the formula d0, and pseudo-bootstrap support values of ≥50 are shown on nodes. Predicted OPTSIL taxon at the family and genus levels are shown as well as the G + C content and sequence length.
